# Prenatal Exposure to DEHP Affects Spermatogenesis and Sperm DNA Methylation in a Strain-Dependent Manner

**DOI:** 10.1371/journal.pone.0132136

**Published:** 2015-08-05

**Authors:** Julien Prados, Ludwig Stenz, Emmanuel Somm, Christelle Stouder, Alexandre Dayer, Ariane Paoloni-Giacobino

**Affiliations:** 1 Department of Mental Health and Psychiatry, Division of Psychiatric Specialties, University Hospitals of Geneva, Geneva, Switzerland; 2 Department of Microbiology and Molecular Medicine, University of Geneva, Geneva, Switzerland; 3 Endocrinology, Diabetes & Metabolism Service of the Centre Hospitalier Universitaire Vaudois and Department of Physiology, University of Lausanne, Lausanne, Switzerland; 4 Department of Genetic Medicine and Development, Geneva University and University Hospital, Geneva, Switzerland; 5 Swiss Center for Applied Human Toxicology, University of Geneva Medical School, Geneva, Switzerland; 6 Department of Basic Neurosciences, University of Geneva Medical School, Geneva, Switzerland; Qingdao Agricultural University, CHINA

## Abstract

Di-(2-ethylhexyl)phtalate (DEHP) is a plasticizer with endocrine disrupting properties found ubiquitously in the environment and altering reproduction in rodents. Here we investigated the impact of prenatal exposure to DEHP on spermatogenesis and DNA sperm methylation in two distinct, selected, and sequenced mice strains. FVB/N and C57BL/6J mice were orally exposed to 300 mg/kg/day of DEHP from gestation day 9 to 19. Prenatal DEHP exposure significantly decreased spermatogenesis in C57BL/6J (fold-change = 0.6, p-value = 8.7*10^-4^), but not in FVB/N (fold-change = 1, p-value = 0.9). The number of differentially methylated regions (DMRs) by DEHP-exposure across the entire genome showed increased hyper- and decreased hypo-methylation in C57BL/6J compared to FVB/N. At the promoter level, three important subsets of genes were massively affected. Promoters of vomeronasal and olfactory receptors coding genes globally followed the same trend, more pronounced in the C57BL/6J strain, of being hyper-methylated in DEHP related conditions. In contrast, a large set of micro-RNAs were hypo-methylated, with a trend more pronounced in the FVB/N strain. We additionally analyze both the presence of functional genetic variations within genes that were associated with the detected DMRs and that could be involved in spermatogenesis, and DMRs related with the DEHP exposure that affected both strains in an opposite manner. The major finding in this study indicates that prenatal exposure to DEHP can decrease spermatogenesis in a strain-dependent manner and affects sperm DNA methylation in promoters of large sets of genes putatively involved in both sperm chemotaxis and post-transcriptional regulatory mechanisms.

## Introduction

In the past century in industrialized countries, a decline in sperm counts has been documented in young healthy men, which could be responsible for the observed decline in fertility [1]. The rapidity of the changes suggested that environmental factors may play a role. Additionally, in regards of the ubiquitous possibility of being exposed to phthalates in our industrialized societies and of the well documented impact of these toxicants on the male reproductive system, it is essential to better characterize, in animal models, the mechanisms of action of an important member of this class of toxicants, Di-(2-ethylhexyl)phthalate (DEHP). End of 2014, the European Chemical Agency classified DEHP in “substances of very high concern” because of its endocrine disrupting properties (ECHA/NA/14/56) and this new verdict could affect the existing authorization applications.

DEHP is a phthalate esters industrial plasticizer found ubiquitously in the environment and strongly suspected to disrupt rodent and human endocrine systems according to various *in vitro* and *in vivo* studies, and one of its metabolite, the Mono-(2-Ethylhexyl) Phthalate (MEHP), was also incriminated. It was observed in rodents that pre- or peri-natal exposure to DEHP induced a decrease in the anogenital distance [2, 3] chryptorchidism [4] and low levels of circulating testosterone [2, 3]. It also induced testes alterations involving Sertoli, Leydig, spermatogenic and spermatogonial stem cells [5, 6], and spermatogenesis alterations [6–9]. In humans, pre- or post-natal exposure to DEHP, as documented in some studies by abnormally high levels of phthalates in the urine or in the semen, induced a reduction in the anogenital distance, an incomplete testicular descent [10] and reduced sperm quality [11, 12].

A functionally important player in epigenetic gene regulation is DNA methylation. It occurs at the level of CpG dinucleotides in specific sites within or around the genes. In imprinted genes, DNA methylation results in an allele-specific silencing of the gene. It also induces a silencing of genes with CpG-rich promoters. Epigenetic changes, in particular DNA methylation, play an important role during development and produces patterns that can persist up to adulthood [13] and even in the next generation [6, 14–16].

Pharmacokinetic studies on orally administrated low dose of radiolabelled DEHP in male mice showed mainly urinary excretion of the radioactivity, but additionally, radioactivity detectable in testis [17]. It has been shown that 2-ethyl-(1-C14)-hexyl-labeled DEHP and mono(2-ethylhexyl) phthalate (MEHP) associate strongly with purified DNA [18]. Previous reports additionally showed increased DNA methylation and increased expression of DNA methyl-transferases in mice testes due to maternal exposure to DEHP [19].

The background hypothesis of the present work, to partially explain DEHP negative effect on spermatozoa production in mice, is that DNA methylation regulating the expression of genes involved in the maturation of sperm cells may be affected by DEHP molecules or metabolites. Additionally, methylation changes might also be involved in the strain-specific sensitivity observed in rodents after exposure to specific environmental factors [20–22].

Here we aimed to investigate the long-term impact of prenatal exposure to DEHP on spermatogenesis and sperm DNA methylation in two distinct mice strains using a genome-wide DNA methylation approach. C57BL/6J and FVB/N mice were selected based on that fact that their imprinting was shown to be modulated by the genetic background [23, 24]. Moreover, the genomes of the two strains are known: C57BL/6J strain corresponds to the usual reference genome whereas FVB/N strain was recently sequenced [25]. Finally, the FVB/N strain has good reproduction capacities with big pronuclei, high litter size and low cannibalization of newborn compared with other strains such as C57BL/6J [26].

In the present study, pregnant C57BL/6J and FVB/N mice were treated orally with either the vehicle (corn oil) or corn oil with DEHP at a dose of 300 mg/kg body weight from day 9 to 19 of pregnancy, i.e. during the time of embryo sex determination [27]. The DEHP dose chosen was in the range of doses at which a reproductive phenotype was observed in rodents [19, 28, 29].

The possible effects of DEHP on the genome-wide methylation patterns were compared between sperm sample of male offspring of C57BL/6J and FVB/N mice, each condition being tested in quintuplicate. The method selected is MBD-Seq and corresponds to high-throughput sequencing using the Illumina technology of methylated DNA fragments previously captured by the methyl-CpG binding domain of MeCP2 protein. MBD-Seq has shown to be a cost-effective and a high resolving approach [30]. That technic allowed measurement of “DNA methylation” on a genome-wide scale, meaning the addition of a methyl group to the 5th carbon position of the cytosine pyrimidine ring by a methyltransferase enzyme. The MBD binds *in vivo* to approximately 12 bp DNA when containing methylated CpG sites [31].

The analysis focused on genome wide differentially methylated regions (DMR). The promoter region is broadly defined in this study as the sequence beginning 2kb upstream and ending 200 bp downstream the first base of the 5’ end of the gene. That definition is not consistent with the true definition of a promoter, but the probed region is expected to contain the main sequences regulating expression as well as start of transcription in a majority of the genes. To our knowledge, no study on phthalate exposure has ever been conducted to address both genetic and epigenetic effects in parallel and on a global scale.

## Materials and Methods

### Ethics Statement

This study was carried out in strict accordance with the Animal Welfare Act and was approved by the Commission d’Ethique de l’Expérimentation Animale of the University of Geneva Medical School and by the Geneva Veterinarian Office (permit reference: G61/3918).

### Mice DEHP exposure

Wild type FVB/N and C57BL/6J mice are purchased from Charles River (L’Arbresle, France). Mice were maintained at the animal core facility of the Geneva University under conventional accommodation. All animal manipulations were monitored using the Python based Relational Animal Tracking system (PyRAT). Two-month-old female mice are naturally mated with male. Females with a copulation plug the next morning are separated in two groups. Bis(2-ethylhexyl)phthalate (Sigma-Aldrich, St. Louis, USA) diluted in corn oil (Sigma-Aldrich, St. Louis, USA) is administered orally by gavage in doses of 300 mg/kg/day for the first group from day 9 to 19 of pregnancy. The second group consists in control mice that receive the vehicle (corn oil) only by gavage from days 9 to 19 of pregnancy. The male offspring of the treated females are sacrificed at 8 weeks of age by cervical dislocation.

### Spermatozoid count

The whole mouse epididymis and the vas deferens is removed and cut into small pieces in a drop of 250 μl of Phosphate-Buffered Saline (PBS) into a Petri dish. The tissues incubate for 10 min at 37°C to release the sperm. PBS drop containing sperm is transferred in new Eppendorf tube and diluted 50 folds. Spermatozoid count is carried out using a hemocytometer. The total spermatozoid count results from the formula: Cells per ml = the average count per square *dilution factor*10^4 (count 10 squares).

### Statistical method

Statistical significance of difference in spermatozoid counts between control and DEHP treated conditions is tested using independent and bilateral two-sample Student's t-test in each of the two strains. Additionally, a power t-test is performed in R. Standards pre-established parameters were 5% type I error and power of 80%. The number of mice effectively analyzed (n) after the data lock point was 8 individuals for each of the four arms of the study. The total spermatozoid counts across groups showed a standard deviation of 3. The delta value of 4.5 million spermatozoa per milliliter was estimated as the threshold to be reached.

### DNA extraction from sperm

For DNA extraction, the mouse vas deferens and epididymis is dissected out, put onto a Petri dish with a droplet of Phosphate-Buffered Saline (PBS), and scored with a razor blade into small pieces to allow sperm to diffuse into the medium. Spermatozoa are released by puncturing the epididymis with a needle. The sample is then transferred into an Eppendorf tube and the fragments were allowed to sediment during 30 min at 37°C. The supernatant is carefully transferred to another tube and this procedure is repeated 3 times. The final supernatant is carefully removed and centrifuged at 6’000 x g for 3 minutes to pellet the sperm. The purity of sperm cell preparations was checked by phase contrast microscopy and preparations containing somatic cells were discarded. The method used here is adapted from a previously publish process [32]. DNA extraction from sperm cells is performed with the QIAamp DNA Micro kit (Qiagen, Hilden, Germany) according to the manufacturer’s protocol.

### MBD-sequencing

10 sperm-extracted DNA samples of males born from DEHP-treated mothers as well as 10 control DNA samples were submitted separately for preparation and MBD-sequencing to the service provider NXT-DX (Gent, Belgium). Each batch includes samples from 5 C57BL/6J and 5 FVB/N mice so that every condition is done in quintuplicate. Samples preparation involve a fragmentation into DNA fragments of ~200bp with Covaris S2, and a capture of methylated fragments with MethylCap kit (Diagenode, Seraing, Belgium). The protein used for the capture consist of the methyl binding domain (MBD) of human MeCP2 which naturally binds methylated CpG *in vivo*, that was fused to Glutathione-S-transferase (GST) containing an N-terminal His6-tag (Diagenode, Cat. No. mbd-001-100).

Libraries are further prepared and amplified for multiplex sequencing following an adapted version of the ChIP-seq protocol. Sequencing is performed on Illumina Hi-Seq 2000 platform. On average, 15M 2x51 read-pairs were sequenced for DEHP libraries, and 12.5M 2x49 read-pairs were sequenced for control libraries. The raw data and the final database have been deposited in NCBI's Gene Expression Omnibus [33] and are accessible through GEO Series accession number GSE67159 (http://www.ncbi.nlm.nih.gov/geo/query/acc.cgi?acc=GSE67159).

### Mapping MBD-Seq reads

Sequenced read-pairs are aligned onto the mouse genome (UCSC mm10) with Bowtie v0.12.7 [34], discarding ambiguous pairs aligning to multiple positions. The mapper generates alignment files in a standard BAM format that are further processed using scripts in the R programming language making use of Bioconductor packages v3.0 to count the number of read-pair aligning onto defined regions of the genome (R Core Team. 2013; Available from: http://www.R-project.org) [35]. Prior to quantification, a filtration of the aligned read-pairs is performed to remove non-concordant ones (i.e. if space between the two reads of the pair is more than 600bp), as well as those aligning to multiple positions on the genome, and those without CpG in their insert (SNPs dependent CpGs are also considered here).

### Probes designs

Two sets of probes were generated *in silico* for this work in order to capture methylation levels. First, so-called “genome-wide-probes” were generated by cutting the mouse genome into 2.2 kb continuous sequences to assess the global distribution of DMRs across the genome. Genome-wide-probes were further assign to the genomic features they overlaps and prioritizing promoter over exons over introns over enhancer over intergenic regions in case of multiple overlaps. Second, so-called “promoter-probes” were arbitrarily defined as the 2.2kb regions ranging from 2kb upstream to 200bp downstream of the start of each gene. All probes were then selected for the analysis if at least one CpG site or one SNP dependent CpG site was present in its sequence and if more than 3 MBD-Seq derived read-pairs were align in more than 2 samples. Enhancers DNA sequences were downloaded from the Vista Enhancer database (http://enhancer.lbl.gov/). We took into account only the 1154 “positive” enhancers that were validated in mouse *in vivo* in “at least three independent transgenic embryos”; 257 were originally identified in the mm9 mouse genome and 897 came from the hg19 human genome. These sequence-based enhancers were mapped onto the mm10 mouse genome with the software BWA, resulting in a total of 836 unambiguously mapped enhancers.

### DNA methylation levels and DMR detection

Methylation level is reflected by the number of read-pairs overlapping the probe after normalization using generalized linear statistical model implemented in EdgeR that has been specifically developed to identify significant statistical differences directly from reads counts [36]. Hyper- and hypo-methylation between Control and DEHP-exposed samples had to show a log2(Fold Change) higher than +1.0 (resp. lower than -1.0). P-value lower than 10^−2^ is required for a DMR identified within genome-wide-probes in which we do not identify specific targets, but rather global picture across genomic features, whereas correction for multiple testing is perform for promoter-probe analysis setting a p-value threshold at 2.2*10^−6^.

### Strain-specific DMR s detection method

For strain-specific DMRs, the EdgeR design considered tests a given region for the following expression to be significantly different of zero:

Z=(C57BL/6J.control−C57BL/6J.dehp)−(FVB/N.control−FVB/N.dehp)

In the formula, *C57BL/6J*.*control* stands for the methylation count of the 5 control samples from C57BL/6J strain, while *FVB/N*.*dehp* stands for the methylation count of the 5 DEHP-treated samples from FVB/N strain. The formula is clearly equal to zero when the difference of methylation between control and DEHP-treated samples is the same in the 2 strains. This test captures DMR showing opposite response to treatment between strains.

### Annotations of the dataset

Gene ontology (GO) terms: “reproduction (GO:0000003)”, “reproductive process (GO:0022414)”, or “sperm part (GO:0097223)” [37]; as well as imprinted genes according to Geneimprint catalog for mouse (http://www.geneimprint.com/) were recorded. Every DMR are further annotated with the variants known between C57BL/6J and FVB/N strains in the vicinity of the region. To establish the genetic background of our mouse strains, we refer to the *Mouse Genome Project* of the Wellcome Trust Sanger Institute [25, 38]. Release v3 of the project establishes a list of variants between 18 mouse strains, including FVB/N and C57BL/6J. From this source, we retrieve homozygous variants of FVB/N strain that differ from C57BL/6J strain with an evidence of at least 3 reads of coverage. This filtration results in 4’931’916 SNPs and 10’525 Indels that distinguish between FVB/N and C57BL/6J strains. Out of those variants, 18’840 modify the protein sequence of the gene they affect. Additionally, RNA expression obtained with sperm cells subpopulations from C57BL/6J males in previous and independent work performed by Chalmel F et al [39] were integrated into the dataset as well as genes whose proteins were identified as part of the sperm proteome of Swiss mice in the work of Baker et al.[40]. The final dataset is publicly available, see the “MBD-sequencing” section.

### Bisulfite pyrosequencing in Tmem125 (6 CpG sites) & Smim8 (1 CpG site)

F1 sperm derived DNA sample of the four conditions tested (C57BL/6J controls and DEHP 300, FVB/N controls and DEHP 300) were analyzed by bisulfite pyrosequencing using six independent samples per conditions except for C57BL/6J DEHP 300 where 2 triplicates were used instead of the six independent samples due to a lake of sufficient remaining samples. DNA was subjected to bisulfite treatment using EZ DNA Methylation-Lightning kit with respect to the manufacturer recommendations (Zymo Research, USA). PCR was performed using the Takara EpiTaq HS (Takara Bio Inc., R110Q). PCR products were immobilized onto streptavidin-coated sepharose beads (Fisher Scientific, ref 17-5113-01) reacting with primer incorporated biotin before being subject to single strands preparation using a Vacuum preparation tool (Biotage) as following the process previously described [41]. The resulting single stranded DNA molecules were then sequenced in a PyroMark Q24 instrument (Qiagen) using appropriate enzymes, substrates and nucleotides form the PyroMark Q24 kit (Qiagen, reference number 970922). Nucleotide dispensation was according to “Method 011” as recommended for the cartridge we receive (Qiagen, catalogue number 979202). Methylation levels at CpG sites were automatically determined by the instrument. Primers and assays related sequences are provided in supplementary (S1 Table).

### Selection of candidate genes for expression measurements

The mRNA expressions between controls and DEHP 300 conditions in both backgrounds (C57BL/6J and FVB/N) were measured by RT-qPCR for a subset of six selected genes; 1) *Star*, 2) *Piwil2*, 3) *Piwil4*, 4) *Rabl2*, 5) *Pacrg* and 6) *Ifg2r*. First, *Star* was selected because of its central role in steroid hormone synthesis and its reported expression modified after DEHP exposure in various organisms [42–50]. *Star* revealed opposite methylation and expression changes upon DEHP exposure in the organism *Oryzias melastigma* [51]. 2) *Piwil2* and 3) *Piwil4* genes were selected based on the report of “epigenetic disruption of the PIWI pathway in human spermatogenesis disorders” [52]. 4) *Rabl2* was selected because of its essential reported role in male fertility, more precisely in the formation of sperm tail [53]. 5) *Pacrg* gene was selected based on its reported deletion causing sterility and its high expression levels in testes in both mice and humans [54]. 6) *Igf2r* was selected because it was the only gene showing imprinting, functional variants and association with all the three Go terms related with the reproduction process in our *in silico* analysis.

### RNA extraction

The mice were placed after sacrifice on carbonic ice and the vas deferens and epididymis were dissected out, placed into a Petri dish, scored with a razor blade in a droplet of Trizol (TRIzol Reagent, Invitrogen, Carlsbad, CA, USA) into small pieces to allow sperm to diffuse in the reagent. The sperm was then transferred in Eppendorf tube with fresh Trizol Reagent and either placed at -20°C or used directly for RNA extraction using the Trizol method [55]. RNA quality was assessed by quantification with a NanoDrop spectrophotometer.

### RT-qPCR

Reverse transcription was performed with Omniscript RT kit (Qiagen, Hilden, Germany) according to the manufacturer’s protocol using 500 ng total RNA. The expression of genes were determined by quantitative real-time PCR using a LightCycler 480 Real-Time PCR System (Roche, Basel, Switzerland) and were normalized using the housekeeping gene *HSP90*. PCR thermal conditions were 10 min at 95°C; then 40 cycles of 10 sec at 95°C, 15 sec at 60°C (for *Star*, *Pacrg*, *Rabl2*) or 55°C (for *Igf2R*) and 10 sec at 72°C. For *Piwil2*: 10 min at 95°C; then 40 cycles of 30 sec at 95°C, 30 sec at 60°C and 30 sec at 72°C. For *Piwil4*: 5 min at 95°C; then 40 cycles of 30 sec at 95°C, 30 sec at 58°C and 30 sec at 72°C. PCR products were quantified using the LightCycler 480 SYBR Green I Master (Roche, Basel, Switzerland). Results are expressed in arbitrary units (A.U) relative to the control group mean value. Primers used to measure expression levels of *Piwil4* and *Igf2R* were designed using the Primer Express software (Applera Europe, Rotkreuz, Switzerland) and provided by Eurofins Genomic (Ebersberg, Germany). Primers for *Piwil2* were described by Lee et al. [56], those for *Star* by Hu et al. [57], those for *Pacrg* by Brody et al. [58] and finally, primers for *Rabl2* by Lo et al. [53]. The sequences of the primers are listed in supporting information (S1 Table).

## Results

### Strain-dependent impact of prenatal DEHP exposure on F1 spermatozoa production

**Fig 1:**
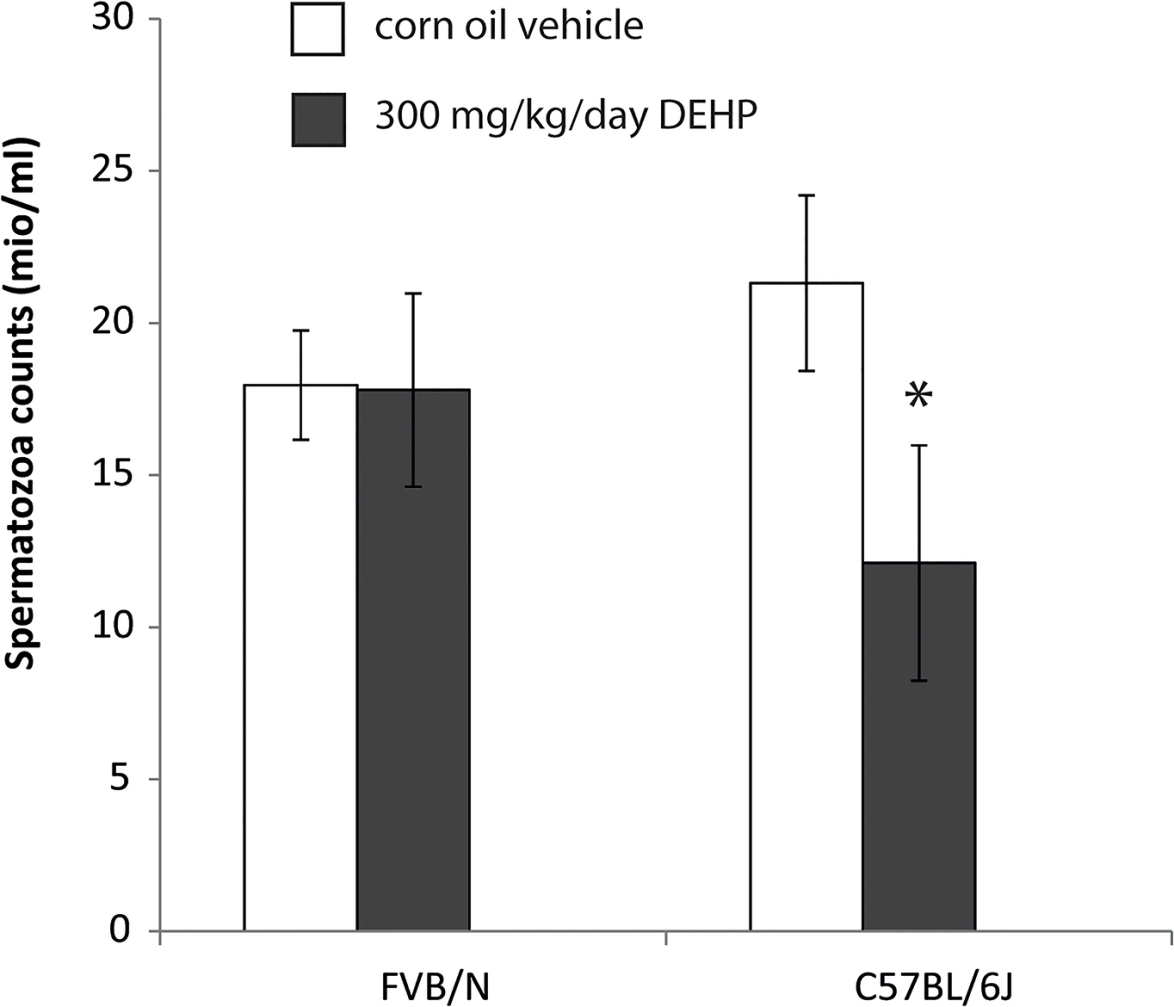
Production of spermatozoa in 8 weeks-old F1 males prenatally exposed to DEHP. Histogram plot showing on the y-axis the spermatozoa count expressed as millions spermatozoa per milliliters (mio/ml) recorded from sperm of F1 males mice, and on the x-axis, the two genetic backgrounds studied with either prenatal exposure to 300 mg/kg/day DEHP diluted in corn oil (gray bars), or corn oil vehicle only (white bars). Prenatal exposure consists of oral administration during pregnancy days 9 to 19 to the pregnant mice childbearing the future F1 males. Errors bars represent standards deviation. *Statistical significant T-test performed between counts recorded in C57BL/6J controls compared with C57BL/6J DEHP treated (Mean C57BL/6J CTL = 21.3, mean C57BL/6J DEHP 300 = 12.1, t = 5, df = 8, p-value = 8.7*10^−4^).

The number of sperm cells expressed as millions spermatozoa per milliliters (mio/ml) decreased in a statistically significant manner upon DEHP exposure at 300 mg/kg/day in C57BL/6J strain as compared with controls (Mean CTL = 21.3, mean DEHP 300 = 12.1, t = 5.043, df = 8.384, p-value = 8.7*10^−4^), and the difference between the means was two-fold higher than the delta threshold of 4.4 determined by the power T-test (Fig 1). No effect of DEHP could be detected on the number of sperm cells in the FVB/N strain at 300 mg/kg/day (mean CTL = 17.96, mean DEHP 300 = 17.80, t = 0.13, df = 11, p-value = 0.9).

### Genomic methylation in F1 males’ sperm DNA

**Fig 2:**
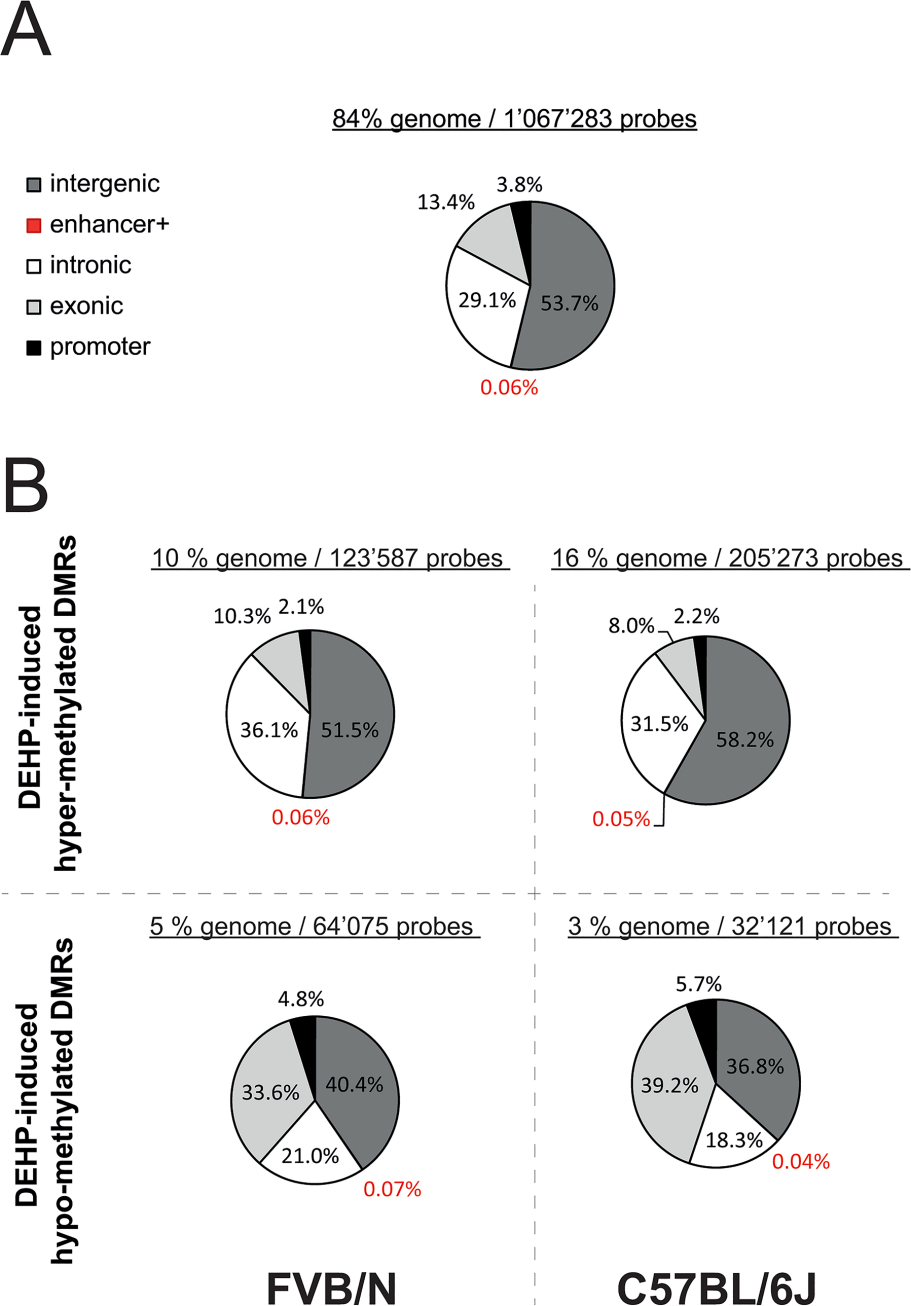
Genomic distribution of sperm-derived DMRs induced by prenatal exposure to DEHP in susceptible and resistant F1 mice. **(A)** Pie chart showing the distribution in % of 2.2 kb sized generated probes among five determined genomic features, exons (light gray), promoters (black), intergenic regions (dark gray), enhancer (red) and introns (white). A total of 1’067’283 starting probes covering 84% of the genome showed an original genomic assignment-based repartition as following; 54% (573'396) intergenic regions, 29% (310’194) introns, 13% (142’843) exons, 0.06% enhancers (684) and 4% (40’166) promoters. **(B)** Pie charts reflecting the relative distribution expressed in percentages of 2.2 kb sized probes among five determined genomic features that showed statistically significant (p-value below 0.01) either two-fold or more hyper-methylation (the two pie charts located above the horizontal dashed line), or two-fold or more hypo-methylation (the two pie charts located under the horizontal dashed line) when comparing DEHP treated mice to controls. Both backgrounds are analyzed separately, FVB/N on the left of the vertical dashed line, C57BL/6J on the right of the vertical dashed line. The number of probes and the coverage in % of the genome is shown for each of the four tested conditions separated by both dashed lines. The repartitions of probes that did not show DMRs in both strains are not shown; were 55% intergenic, 29% intronic, 12% exonic, 4% promoter and 0.06% enhancer in FVB/N and respectively 53%, 29%, 14%, 4% and 0.06% in C57BL/6J strains. These repartition of probes not affected by DMRs respected the original repartition of probes (A).

Whole genome analysis of DNA sperm methylation using MBD-Seq indicated that prenatal exposure to DEHP has a higher impact on the fraction of hyper-methylated DMRs in the C57BL/6J strain compared to the FVB/N strain (16% compared with 10%) and less impact on the fraction of hypo-methylated DMRs (3% compared with 5%, respectively) (Fig 2). More specifically, following DEHP-exposure 16% of probes were hyper-methylated and 3% were hypo-methylated in the C57BL/6J strain as compared to 9.7% and 5% respectively in the FVB/N strain. DEHP effects were not randomly distributed in the genome with a secondary finding of approximately three times more exons-assigned probes within hypo-methylated DMRs (34% in FVB/N and 39% in C57Bl/6J compared with 13% at the origin) combined with approximately two times less promoter-assigned probes within hyper-methylated DMRs in both backgrounds (2% in both backgrounds compared with 4% at the origin).

### Prenatal DEHP exposure increases promoter methylation of vomeronasal and olfactory receptors genes and decreases promoter methylation of microRNAs

**Fig 3:**
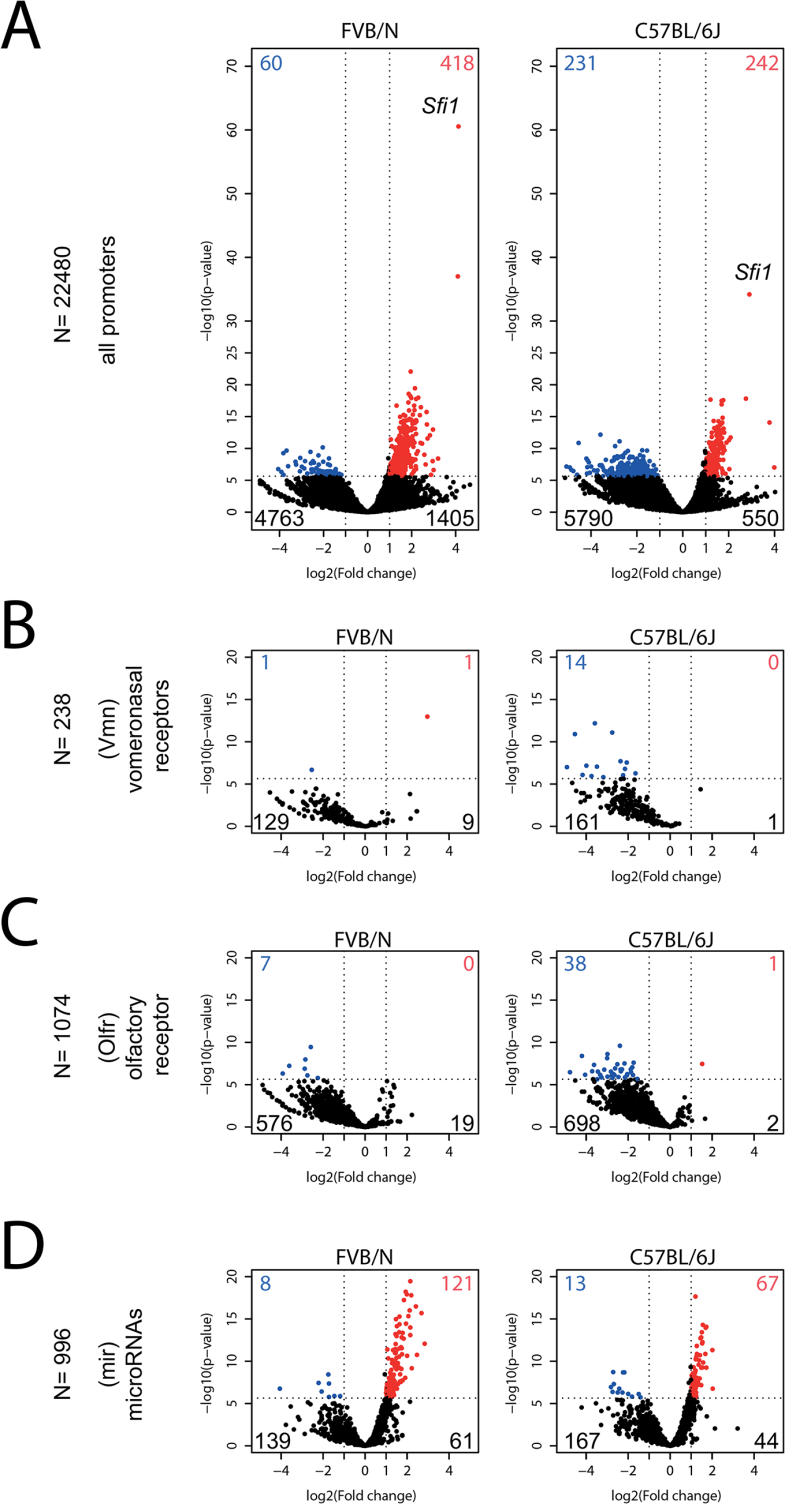
DEHP-induced DMRs in sperm promoters. Volcano plots illustrating promoter methylations in function of the prenatal exposure to DEHP in sperm in both backgrounds. The data were obtained from 5 control mice compared with 5 DEHP treated mice for each background, FVB/N shown left and C57BL/6J shown right. X axis represent the log_2_ fold change between controls and DEHP. Y axis represents the minus log_10_ of the p-value. Horizontal dashed lines represent the genome-wide statistical significance level settled at 2.2*10^−6^ (0.05 alpha divided by 22’480 tested probes). Vertical lines represent biological significance thresholds for DMRs settled above 1 and under -1 on the x-axes, corresponding respectively to two-fold methylation increase in controls compared with DEHP-treated and two-fold methylation decrease in controls compared with DEHP-treated. Blue points represent promoter showing statistically and biologically significant increased methylations in DEHP, whereas red points represent promoter showing statistically and biologically significant increased methylation in controls. The total of blue and red points are indicates on each graph using blue and red numbers. The number of promoter showing a statistically not significant more than two-fold increase methylation level in DEHP condition is indicates in black on the bottom left of each graph, whereas the number of promoter showing a statistically not significant more of than two-fold decreased methylation in DEHP compared to control is indicates in black on the bottom right of each graph. (**A)** Volcano plots of methylation results based on normalized reads numbers that were recorded in 2.2kb sized probes covering 22’480 different promoters, each assigned to a different murine gene. The most significant point corresponds to the promoter of *Sfi1* and is indicated. (**B)** Volcano plots of methylation results based on normalized reads numbers that were recorded in 2.2kb sized probes covering a total of 238 vomeronasal receptors whose gene symbols contains “Vmn”. (**C)** Volcano plots of methylation results based on normalized reads numbers that were recorded in 2.2kb sized probes covering a total of 1074 olfactory receptors whose gene symbols contains “Olfr”. (**D)** Volcano plots of methylation results based on normalized reads numbers that were recorded in 2.2kb sized probes covering a total of 996 micro-RNAs whose gene symbols contains “Mir”.

The number of promoters significantly methylated in DEHP-exposed mice shown as blue points was higher in the C57BL/6J strain (n = 231) compared to FVB/N (n = 60), whereas more de-methylated promoters appearing as red dots were presents in FVB/N (n = 418) compared to C57BL/6J strain (n = 242), (Fig 3A). The most significant DMR recorded was *Sfi1* promoter hyper-methylated in controls in both backgrounds (Fig 3A, highest red points in both volcano plots). Three main groups of genes were identified by high occurrences of gene names beginning with “*vmn*”, “*olfr*” and “*mir*” in DEHP-induced significant DMRs. The two first consist of genes encoding vomeronasal (Vmn) and olfactory (Olfr) receptors. Massive and global trends to be hyper-methylated in DEHP conditions were observed for 55% ((129+1)/238) of all the *vmn* promoters in FVB/N and respectively for 74% ((161+14) /238) in C57BL/6J, with similar trends for 54% ((576+7)/1074 of all *olfr* genes in FVB/N and for 69% ((698+38) /1074) in C57BL/6J, whereas bucking events comparatively remains rare (n = 10, 1, 19 and 3) (Fig 3BC). Higher numbers of those significant targets are observed in C57BL/6J strain compared to FVB/N, respectively 14 and 38 compared with 1 and 7 (Fig 3BC). DEHP conditions had more pronounced statistically significant and opposite impacts on micro-RNAs’ promoters, this class represents almost a third of all the significant hypo-methylated genes in both strains (Fig 3AD) with a higher number of significant hypo-methylated promoter in FVB/N compared to C57BL/6J, 121 versus 67 (Fig 3D).

### Strain-specific DEHP-induced DMRs

**Fig 4:**
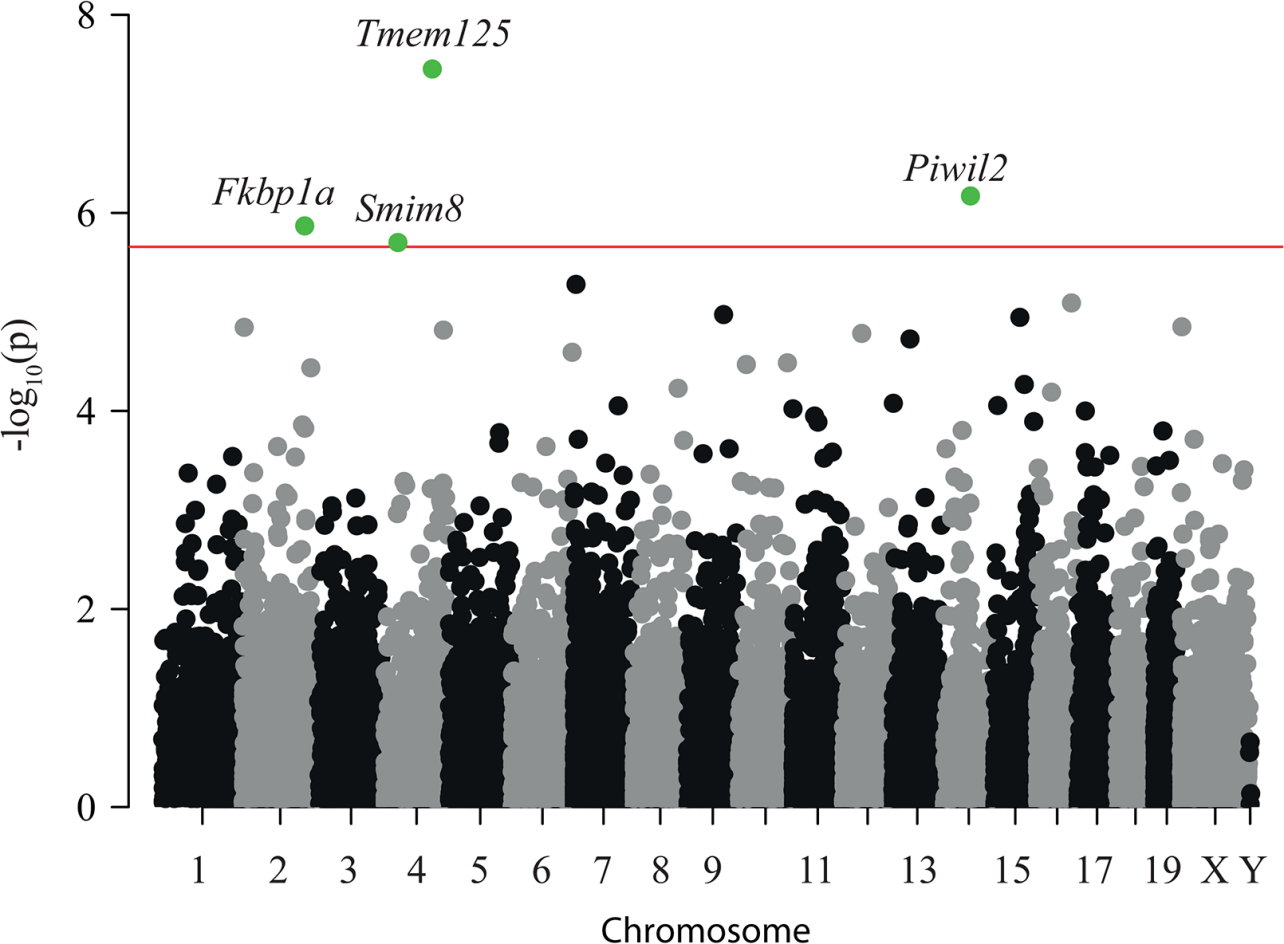
Strain-specific DEHP-induced DMRs. The Manhattan plot shows the statistical significance in minus log10 on the y axis, and the location of the probes in the genome with alternate gray and black colors in respect to the chromosomes locations on the x-axis. Horizontal red line correspond to the threshold for statistical significance settled as p = 0.05 corrected with the number of tested probes (yielding p<2.2*10^−6^). The name of the gene is given and the point appears green in case of genome-wide statistical significance. 5 individuals were tested in the four groups (FVB and C57BL/6J background with both control and DEHP300 treatment).

Strain-specific DEHP-induced DMRs correspond to promoter in which hypo or hyper-methylation in DEHP condition compared to control is observed in one of the two tested strains, whereas opposite or no impact is observed for the same promoter when tested in the other strain. These strain-specific DMRs were analyzed by testing the following formula to be statistically different from zero: *Z* = (*C*57*BL*/6*J*.*controls* − *C*57*BL*/6*J*.*dehp*) − (*FVB*/*N*.*controls* − *FVB*/*N*.*dehp*). Results visualized in a Manhattan plot showed four targets differently affected in term of methylation by DEHP in function of the tested strain and with p-values below the genome wide significance threshold of p < 2*10^−6^: 1) *Tmem125* [log2(FC) in C57BL/6J = 3.8, log2(FC) in FVB/N = 0.2, FDR = 0.0008, p = 3.54*10^−8^], 2) *Piwil2* [log2(FC) in C57BL/6J = -1.3, log2(FC) in FVB/N = 1.5, FDR = 0.0075, p = 6.72*10^−7^], 3) *Fkbp1a* [log2(FC) in C57BL/6J = -4.2, log2(FC) in FVB/N = 2.7, FDR = 0.0101, p = 1.35*10^−6^] and 4) *Smim8* [log2(FC) in C57BL/6J = -4.1, log2(FC) in FVB/N = 3.6, FDR = 0.0113, p = 2.00*10^−6^] (Fig 4). Therefore, *Tmem125* promoter was hypo-methylated in DEHP compared with control conditions in C57BL/6J strain, whereas no notable methylation changes could be detected when testing the other FVB/N strain. *Piwil2*, *Fkbp1a* and *Smim8* promoters were hyper-methylated in DEHP compared with control conditions in C57BL/6J strain, and hypo-methylated in DEHP compared with control conditions in the other tested strain FVB/N. These four targets were indeed DEHP-dependent; they did not differ significantly when compared between strains in controls (S1 Fig).

### Confirmatory bisulfite pyrosequencing experiment in Tmem125 & Smim8

**Fig 5:**
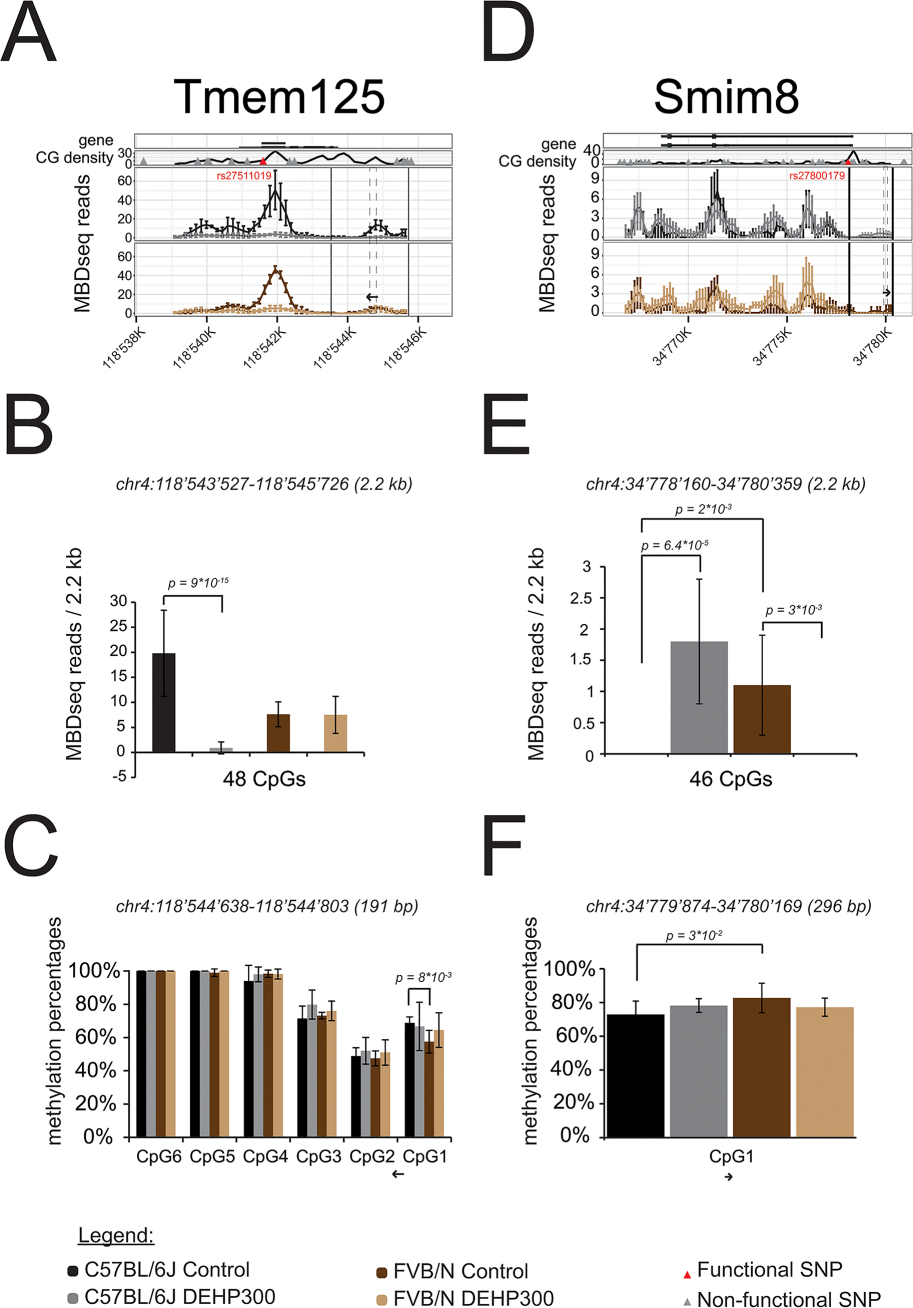
Confirmatory bisulfite pyrosequencing experiment in Tmem125 & Smim8. **(A, B, C)** Images related with *Tmem125*. (**D, E, F)** Images related with *Smim8*. (**A, D)** Full scan derived from MBDseq data reflecting methylation levels in the entire genes and with the promoter regions as defined in the manuscript for the four tested conditions. The densities of CpG throughout the entire gene are shown as well as SNP between strains. The legend is located at the bottom of Fig 5 and the colors encoding the four conditions are respected all over the Fig 5. (**B, E)** Promoter methylation according to MBDseq experiments probing 2.2 kb promoters delimited and localized with vertical lines on the full scan in (**A**) and (**D**). (**C, F)** Promoter methylation according to pyrosequencing experiments probing the regions defined by dashed line on the full scan shown in (**A**) and (**D**) with an arrow indicating the sense of the pyrosequencing experiment. P-values resulting from T-Tests performed between conditions smaller than 0.05 and coordinates of the probed regions according to the mm10 mouse genome are shown (**B**, **E**, **C**, **F**).

Result of the confirmatory pyrosequencing experiments that were performed for the two unknown targets *Tmem125* and *Smim8* in order to validate the opposite promoter methylation impacts between strains upon DEHP exposure is summarized on (Fig 5). Pyrosequencing derived methylation percentages obtained for CpG_1_ in *Tmem125* promoter were statistically significantly higher (p = 8*10^−3^) in C57BL/6J controls (69±4%) compared to FVB/N controls (58±7%), (Fig 5C), which is consistent with MBDseq results in C57BL/6J controls (19.8±3.9) compared to FVB/N controls (7.6±2.5), although that time not statistically significant (p = 0.11), (Fig 5B). Inversely, a statistically highly significant DEHP-induced decreased reads numbers (p = 9*10^−15^) was observed in *Tmem125* promoter region in MBDseq experiment in C57BL/6J controls (19.8±3.9) compared with DEHP300 (0.9±3.9%), (Fig 5B), consistent with a DEHP-induced decreased methylation levels recorded in pyrosequencing experiment in CpG_1_ although not statistically significant (p = 0.7) in C57BL/6J controls (69±4%) compared to DEHP300 (65±10%), (Fig 5C). Note that in the *Tmem125* assay probing 6 sites, we do not consider CpG_2–6_, because they go away form the MBDseq pic detected (Fig 5A) and because pyrosequencing is prone to inaccuracy in distant site. In *Smim8*, a statistically significantly lower methylation level was recorded by pyrosequencing experiment in CpG_1_ (p = 3*10^−2^) in C57BL/6J controls (73±8%) when compared with FVB/N controls (83±5%), Fig 5F, which is consistent with an also statistically significant lower average of reads (p = 2*10^−3^) that was observed in C57BL/6J controls (0±0) when compared with FVB/N controls (1.18±0.8), Fig 5E. Inversely, both statistically significant and opposite impacts between strains of the DEHP treatments on methylation levels observed in MBDseq experiments (Fig 5E) are concordant with same trends although not statistically significant observed in pyrosequencing experiments (Fig 5F). Indeed, MBDseq resulted in an increased reads averages recorded in DEHP 300 (1.8±1) compared with those recorded in controls (0±0) in C57BL/6J background (p = 6.4*10^−5^), and a decrease reads in DEHP 300 (0±0) compared with controls (1.1±0.8) in the other FVB/N background (p = 3*10^−3^), (Fig 5E), consistent with pyrosequencing derived increased methylation levels recorded in DEHP300 condition (78±9%) compared with controls (73±8%) in C57BL/6J background and a decrease methylation level in DEHP 300 (77±7%) compared with controls (83±5%) in FVB/N background, (Fig 5F).

### Methylation of promoters correlated with low expression levels

**Fig 6:**
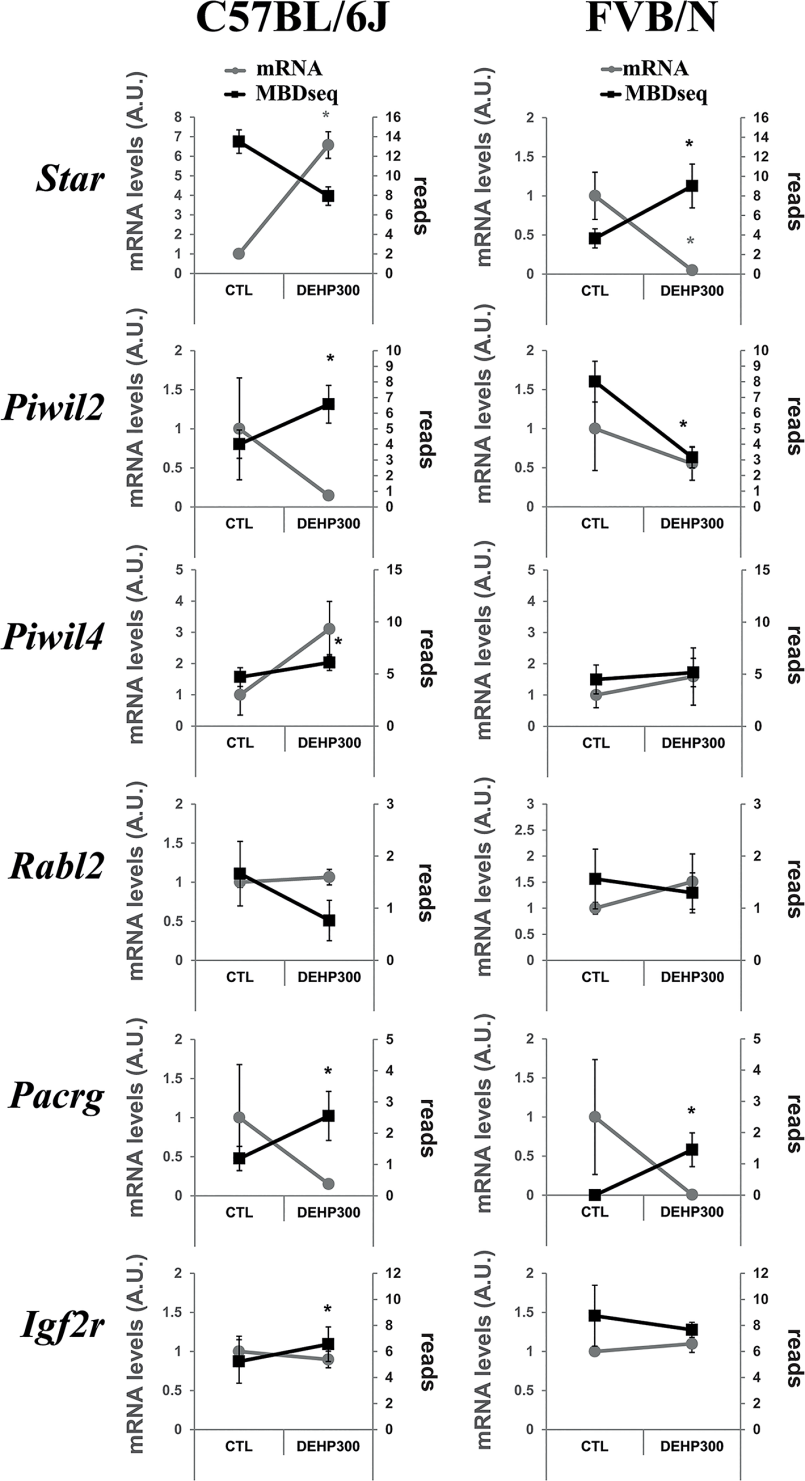
Impact of promoter methylation on gene expressions in pre-selected candidates. Graphical representation of the obtained MBDseq and RT-qPCR results on a subset of pre-selected genes and in respect with the mouse strain background, C57BL/6J on the left part of the figure and FVB on the right side of the figure. The x-axis showed results obtained between the control condition (CTL) always on the left and the DEHP 300 condition (DEHP) always on the right of each graphic as mentioned. Levels of mRNA appear as gray dots and are expressed as arbitrary units on the left-side Y-axis labelled in gray and named mRNA levels (A.U.). A.U.: Arbitrary units relative to the control condition. The MBD-seq promoter methylation levels appear as black squares and are expressed in average of reads for each condition labelled on the right-side Y-axis in each graphic. Errors bars represent standard error of measurements for mRNA levels and standards deviation for MBD-seq derived reads averages. Inverted slopes between MBDseq and mRNA data are interpreted as results consistent with a negative impact of promoter methylation on gene expression. Stars in gray represent significant differences in term of mRNA levels obtained between controls and DEHP 300 conditions according to two-tails T-test resulting in p-value lower than 0.05. Stars in black represent statistically significant differences in term of average reads numbers obtained between controls and DEHP 300 conditions according to two-tails T-test resulting in p-value lower than 0.05.

Expression and methylation in the six pre-selected targets mentioned in the method section are presented on (Fig 6). Results showed inverted slopes between MBDseq and mRNA data suggesting a negative impact of promoter methylation on gene expression.

## Discussion

In this study, we prenatally exposed two different strains of mice to 300 mg/kg/day DEHP at days 9 to 19 of pregnancy, and recorded a statistically significant F1 sperm count decrease phenotype in the C57BL/6J, consistent with previous reports in rodents [6, 7, 9], whereas studies performed in men showed correlation between phthalate exposures and semen quality [11, 12]. By contrast, the production of spermatozoa in FVB/N strain was not affected at that dose, which is in the range of doses for which a reproductive phenotype was observed previously in KM mice 500 mg/kg/day [19, 29] and Sprague-Dawley rats 300mg DEHP/kg/day [28]. It is noteworthy that a two-time higher dose of DEHP resulted in decreased spermatogenesis also in the resistant strain FVB/N, data not shown. The dose of 300 mg/kg/day presently used through oral administration is in this study is several orders of magnitude more elevated than the Environmental Protection Agency (EPA) reference dose of 0.02 mg/kg/day (http://www.epa.gov/ttnatw01/hlthef/eth-phth.html). Doses used for prenatal or lactational exposition in recent animal studies range from 20μg/kg/day to 750mg/kg/day [28, 59–65]. In contrast to direct toxic action, it should be noted that we here investigated a potentially transgenerational effect. DEHP orally administered to dams should cross various biological barriers before reaching fetuses gonads (including digestive tract of the dams, blood maternal circulation, placental barrier, blood embryonic circulation, embryo hematotesticular barrier) leading us to use a high dose (more typical to a toxicological risk assessment study) for this first ‘‘proof of concept” study. Further studies involving weaker doses more relevant of the environmental exposure are needed in the future.

In order to explain the impact of DEHP on spermatogenesis, we hypothesized that DEHP could enter the fetal mouse body during testis development and have a long term effect on the expression of genes involved in spermatogenesis by affecting DNA methylation in immature sperm cells. This hypothesis was supported by a number of studies reporting epigenetic changes induced by endocrine disruptors some reporting trans-generational impacts [14–16, 66, 67].

A first screening of DEHP-induced sperm DRMs within genomic features, presented on Fig 2, was performed to understand how DMRs distribute in the mouse genome. The first main result is that C57BL/6J strain is more affected by both DEHP-induced hypo- and hyper-methylations as compared to FVB/N strain, what seems consistent with the susceptible phenotype in terms of the decreased sperm count we observed in C57BL/6J only (Fig 1). Moreover, the fact that the genomic distribution of hypo-methylated probes, Fig 2B-down, differs from the original distribution of the probes as shown in Fig 2A, suggests that DEHP effects are not randomly distributed in the genome.

We further analyzed methylations in promoters, because of their implications in gene silencing, despite the fact that the global picture suggests that methylations in other genomic features such as exons could also be of interest. That approach reveals a major finding; three functional classes of genes show robust changes in their promoter methylation as shown in Fig 3. The main statistically significant group of de-methylated genes promoters in DEHP treated mice is that of micro-RNAs (Fig 3D), with a more enriched fraction in the resistant FVB/N strain. It was previously shown that microRNA expressed in sperm cells can be delivered into the oocytes during fertilization, but the function of sperm microRNAs remains poorly understood [68]. In more detailed, our observation in mice showed that prenatal exposure to DEHP resulted in decrease spermatogenesis (Fig 1), associated with large pool of micro-RNAs promoters with decreased methylation (Fig 3D).

Interestingly, two clusters of microRNAs called mir-17-92 and mir-106b-25 were reported to promote survival and proliferation of spermatogonia in mice [69]. The first cluster “mir-17-92” is composed with 5 microRNAs; mir17, mir18, mir19a, mir20a and mirhg, all located closed one to each other’s on the chromosome 14. The second cluster “mir-106b-25” is composed with 3 microRNAs; mir25, mir93 and mir106b, all closely located on chromosome 5. In our data, genome-wide significant higher methylation are observed in the resistant FVB/N strain controls when compared to DEHP-treated for all the 8 microRNAs belonging to both mir-17-92 and mir-106b-25 clusters; p-values ranging from 1.8*10^−8^ to 1.1*10^−15^, and log2(FC) ranging from 1.5 to 2.8, whereas only mir17 was significant in the sensible strain C57BL/6J (log2(FC) = 2, p-value = 1.8*10^−7^). The causative relationship between the observed DMRs in microRNAs and spermatogenesis defects is not known, but “increasing evidence supports the essential role of small RNA-mediated RNA regulation in normal spermatogenesis and male fertility” [70], whereas *de novo* RNA-directed DNA methylation (RdDM) pathway described in plant and involved in transposon silencing of plant sperm cells was previously reported [71].

Additionally, two main identified groups of genes coding vomeronasal and olfactory receptors, that were originally discovered in the nose, showed hyper-methylation in sperm of DEHP treated mice, with a higher number of statistically significant targets recorded in the susceptible C57BL/6J strain (Fig 3BC). Even if this could be quite surprising, scientific literature revealed that olfactory receptors are expressed in sperm cells most probably for chemotaxis purposes [72, 73]. The DEHP induced hyper-methylation of *Sfi1* is remarkable because the effect is maximal as compared to other gene promoters in both genetic backgrounds. Sfi1 is a component of centrosomes, conserved from yeast to human [74], and was essential for controlling the process of one centrosome duplication per cell during mitosis in fission yeast [75]. The function of *Sfi1* in sperm remains unknown.

We decided not to discuss in details other significant DMRs that do not belong to the three mentioned classes of genes as well as the extensive analysis we performed on significant DMRs taking in account SNPs between strains, for simplification purposes.

The second approach target DEHP induced DMR that differ between strains. Results showed four genome-wide statistically significant DMRs with opposite DEHP-induced methylation changes observed between the two tested strains: first, *Tmem125* promoter, hypo-methylated in DEHP compared with control conditions in strain C57BL/6J, was statistically significant at the genome level (p = 3.54*10^−8^) in term of a different impact when performing the same comparison in the other tested strain FVB/N, in which no methylation changes occurred between control and DEHP conditions. Second, three other promoters were statistically significant at the genome level, with hyper-methylation in DEHP compared with control conditions in C57BL/6J strain and opposite hypo-methylation for the same comparison performed in the other tested strain FVB/N: *Piwil2* (p = 6.72*10^−7^), *Fkbp1a* (p = 1.35*10^−6^) and *Smim8* (p = 2.00*10^−6^). All those targets are highly expressed and in all tested sperm cell sub-populations according to the public dataset of Chalmel *et al*. [39], with the exception of a low level of expression for *Tmem125*. *Tmem125* and *Smim8* code for membrane proteins of unknown functions. Not a single report could be detected in the Pubmed database with “Tmem125” or “Smim8” keywords. The “P- element Induced WImpy testis-like” *Piwil2 gene*, was previously shown as hyper-methylated in the testicular samples of human infertile males [52], whereas mouse model deleted for *piwil2* (Mili) were sterile [76], suggesting that both the methylation status and the presence of the gene seems important for fertility. The *Piwil1*, *Piwil2* and *Piwil4 genes* are specifically expressed in germline cells [76, 77]. “Two distinct waves of piRNA production” involving the activities of MILI (*Piwil2*) and MIWI2 (*Piwil4*) have been previously reported “during the early stages of mammalian male gametogenesis” [78]. These genes are involved in a complex pathway to repress the expression of transposable elements in the gonads influencing the epigenetic state of transposons [79, 80].

*Fkbp1a* gene product FKBP12 was previously shown to be released in the male reproductive tract and to stimulate sperm motility [81]. More generally, FKBP12 belongs to the FKBP family of proteins in which different members were shown to be essential for fertility. FKBP52 compromises sperm fertilizing capacity [82], FKBP6 is essential for male fertility [83] and was identified as a susceptibility locus for impaired acrosome reaction in stallions and FKBP36 plays a crucial role in male meiosis [84]. Thus, *Piwil2* and *Fkbp1a* might putatively play a role in the strain-specific sensitivity of C57BL/6J spermatogenesis to DEHP exposure.

Among the four targets showing differential DEHP impacts between strains, functional variants were identified in *Tmem125* (rs27511019) and *Smim8* (rs27800179) as well as variation in their promoters. In the *Tmem125* promoter containing 48 CpG sites, the 3 SNPs did not affect CpGs (rs32827594, rs32428625, rs32430621). In the *Smim8* promoter of containing 46 CpG sites, two CpG sites depend of two SNPs (rs265964855, rs31794755), CpG sites being present in C57BL/6J strain and absent in the FVB strain, whereas one SNP (rs27800177) resulted in a CpG site in FVB/N strain only, the 5 others did not affect CpGs (rs33891805, rs48836414, rs242357999, rs32407938, rs32188526). Neither functional variants nor variations in the promoter could be identified within *Piwil2* and *Fkbp1a*. Opposite impacts between strains of the DEHP treatments on *Tmem125* and *Smim8* promoters methylation levels were observed in MBDseq experiments (Fig 4) and partially confirmed by targeted pyrosequencing (Fig 5).

The fact that one strain resists and the other is susceptible, *i*.*e*. sperm count in Fig 1, allowed distinguishing DMR related with the phenotype, “putative functional”, from those that, *a priori*, should not impact the production of spermatozoa. Note that the link between DMRs and phenotype is not achieved here. The most promising putative functional DMRs according to the analysis and interpretation of the data remains those appearing significant in the Manhattan plot in Fig 4. At the opposite DEHP-induced DMR that behaves in a similar manner between both strains should not be directly related with the observed sperm count decreased phenotype. The strain-specific impacts of DEHP on DNA methylation might be due to an alteration of enzymes involved in DNA *de novo* methylation, methylation maintenance and de-methylation. The results of this study suggest that DEHP prenatal exposure affects methylation of the promoters of genes functionally involved in DNA methylation. MBD-Seq results (S1 Dataset) show a DEHP associated decrease of *Tet1* promoter methylation, statistically significant only in the FVB strain (23.6 versus 6.6 mean normalized reads, log2(FC) = 2.2, p = 6.2*10^−8^). They also show a decrease of *DnmT1* promoter methylation statistically significant only in the C57BL/6J strain without correcting for multiple testing (32.9 versus 13.9 mean normalized reads, p = 0.009). These observations are compatible with a previous work performed in mice prenatally exposed to a higher dose of DEHP (500 mg/kg/day) in which significant increases of DNA methyltransferase expressions were reported [19]. Additionally, 2 splice variants (rs47298447 and rs29741090) were reported between both tested strains in *Dnmt1*. It is the first time, in our knowledge, that strain-specific and strain consistent impact of DEHP on sperm methylome has been investigated, allowing an overview of alterations detectable in the sperm methylome after DEHP prenatal exposure. We show that DEHP acts deleteriously at a large scale but not at random. Targets can be identified some with functional variants between both strains that might be involved in the process of DEHP-induced alterations in C57BL/6J mice spermatogenesis. DEHP-induced promoter methylations increased was associated with lower expressions in the selected targets, Fig 6, among which only *Star* was significant for both methylation and expression differences. These results suggest that methylation of promoters induced by DEHP exposure can be functional, such as for *Star*. At the genome-wide scale, genes expression data from sperm cells subpopulations extracted from C57BL/6J males in previous and independent work performed by Chalmel F *et al* [39] was negatively correlated with promoter methylation deriving from our study. The analysis of the converged dataset revealed decreased promoters methylation in mature spermatozoa of genes highly expressed in immature sperm cells, suggesting a time delay between methylation and expression, whereas the proportion of targets whose protein products were identified as part of the proteome in Baker *et al*., 2008 decreased in function of promoter methylations (S4 Fig).

Limitations in the study were the absence of evaluation of sperm morphology and motility, of information related with hydroxy-methylation in sperm DNA, of the impacts of the DMRs on gene expressions and of analysis of micro-RNAs targets. In the sample process, we avoid batch effect impacting strains comparisons, but potential batch effect could impacts comparison performed between controls and DEHP conditions. Difficulties encountered in that study were low amount of material extractable from sperm samples and complex data analysis process.

## Supporting Information

S1 ARRIVE Checklist “The ARRIVE Guidelines Checklist” for reporting animal data completed for this study. (PDF)

S1 Dataset Publically available database containing MBDseq results accessible online with the GEO Series accession number GSE67159 at http://www.ncbi.nlm.nih.gov/geo/query/acc.cgi?acc=GSE67159 with an associated file GSE67159_S1_dataset_description. (DOCX)

S1 Fig MBD-seq sperm-derived analysis of promoter methylation differences between C57BL/6J and FVB/N mouse strains. **Left**, scatter plot presenting analysis of MBDseq derived reads mapping promoters and comparing both strains under control conditions. Each point corresponds to one of the 22’480 probed promoters tested sized of 2.2 kb. Significant strain-specific DMRs shown as either blue or black points were identified with both p-values lower than 2.2*10^−6^ and more than two-fold difference between strains in both directions. None of the four targets identified in Fig 4 (*Tmem125*, *Piwil2*, *Fkbp1a* and *Smim8*) differs significantly in methylation between strains, (red labelled points). DMRs analysis was performed following Edge-R. Results: 28 promoters were significantly more methylated in FVB/N (blue points). 78 promoters were significantly more methylated in C57BL/6J (black points). 69 promotes had highly similar levels of methylation in both strains (yellow points), as shown by p-values, deriving from the strain-specific DMRs detection method, higher than 0.995. Those points should correspond to some of the lowest points that were presented on the y axis in the Fig 4. They were additionally selected by presenting more than 1 reads in means for both strains. **Right**, scatter plot presenting analysis of MBDseq derived reads mapping promoters and comparing both strains under DEHP conditions. By comparing with the controls on the left, it can be seen that the 78 promoters that were significantly more methylated in C57BL/6J as compared with FVB/N controls (black points), still present more reads in C57BL/6J as compared with FVB/N under DEHP condition. Out of the 28 promoters that were significantly more methylated in FVB/N as compared to C57BL/6J controls (blue points), 22 are more methylated, whereas 6 became less methylated in a non-significant manner in strain FVB/N. The 69 yellow points remain in the center of the new cloud, without any notable change of behavior between both comparisons. In the four targets identified (*Tmem125*, *Piwil2*, *Fkbp1a* and *Smim8*), the differences in methylation between strains are significantly different when the test is performed between controls as compared to the same test performed between DEHP conditions. Gene symbols of the 78 targets shown as black points and that were significantly more methylated in C57BL/6J controls compared with FVB/N controls: *Sparc*, *Ankrd66*, *Aldh3a1*, *Zp1*, *D730045A05Rik*, *Rbbp8*, *Ano2*, *A430105I19Rik*, *Mir1936*, *Ugt1a1*, *Kcnj9*, *Pwwp2b*, *Olfr462*, *4921517D22Rik*, *Ddost*, *Tspo2*, *Calr4*, *Hdac9*, *Eps8l1*, *4921524J17Rik*, *1700055C04Rik*, *Ltk*, *Klf2*, *Fgf1*, *Gm5523*, *Prss27*, *B430010I23Rik*, *Gdf15*, *Siva1*, *1700054M17Rik*, *Mroh2a*, *Epb4*.*1l1*, *Cela3a*, *Enpp4*, *Trpm8*, *Cilp*, *Traf6*, *Dpys*, *Kcnk15*, *Fam166b*, *Emp1*, *Kdf1*, *Olfr1033*, *Rgs9*, *Scd3*, *Plekhb1*, *G530011O06Rik*, *Qpct*, *Cmbl*, *Gm20110*, *Dhx34*, *H2-K2*, *Smco3*, *Insl3*, *Gm136*, *Ulbp1*, *Hddc2*, *Zfp637*, *AY761185*, *Gm15698*, *AA388235*, *Sirpb1a*, *C330011F03Rik*, *Apol10a*, *Trim34b*, *Mir6388*, *Mrgprb8*, *Gm9833*, *Pafah2*, *Vmn2r44*, *Cd200r2*, *Afg3l1*, *Tmem181b-ps*, *Ap1m1*, *Gm15284*, *Stk38l*, *Actr2*, *Vmn1r1*. Gene symbols of the 28 targets shown as blue points and that were significantly more methylated in FVB/N controls compared with C57BL/6J controls: *Ceacam-ps1*, *Gna13*, *Dusp13*, *6820431F20Rik*, *Samd8*, *Gm7538*, *Mir6385*, *2810429I04Rik*, *2610005L07Rik*, *5430440P10Rik*, *3110070M22Rik*, *Wfdc9*, *Clec2f*, *Atp11b*, *Olfml3*, *4930481A15Rik*, *Capn11*, *Gm13286*, *Mir466d*, *Scn5a*, *Rnu7*, *Polr3h*, *4932413F04Rik*, *Olfr279*, *Vmn2r29*, *Rps4l*, *Mir6991*, *Rhox3c*. The 6 last targets were not consistent in the comparison performed between strains subjected to DEHP prenatal exposure. Gene symbols of the 69 targets shown as yellow points whose impacts of DEHP on differential methylation levels as compared to controls were similar between both strains: *1500015O10Rik*, *4833417C18Rik*, *4930500F04Rik*, *9330159F19Rik*, *Abca17*, *Acot5*, *Acrbp*, *Atad3a*, *BC003965*, *BC030336*, *Birc3*, *Camk4*, *Chl1*, *Chrm5*, *Cnih1*, *Cntn6*, *Crybg3*, *Ctsl*, *Egln2*, *Enam*, *Enpep*, *Epha8*, *Exoc8*, *Fam134c*, *Fcer1g*, *Gm10451*, *Gm16294*, *Gm53*, *Gnptg*, *Gpr15*, *Hoxd10*, *Klc2*, *Klk1b21*, *Krt14*, *Lrrc4b*, *Mansc4*, *Mir149*, *Mir1899*, *Mir6395*, *Mrgprg*, *Mtor*, *Olfr1306*, *Olfr656*, *Omg*, *Os9*, *Pigq*, *Pkn1*, *Prdx6b*, *Prg3*, *Ptgr1*, *Rasal3*, *Rnaseh2b*, *Sbf1*, *Serpine2*, *Slc22a1*, *Slc25a10*, *Spatc1*, *Tff2*, *Tgif1*, *Timm22*, *Tnfsf13b*, *Uevld*, *Vps8*, *Wdr43*, *Wipf2*, *Wnt3*, *Zfp319*, *Zfp459*, *Zfp62*.
(EPS)

S2 Fig DEHP-induced DMRs in micro-RNAs and their targets. Scatter plots of promoter methylation changes in microRNAs and their targets in function of the prenatal exposure to DEHP and in F1 derived males sperm cells for both backgrounds. The data were obtained from 5 control mice compared with 5 DEHP treated mice for each background. C57: results obtained with strain C57BL/6J presented in the scatterplot on the left. FVB: results obtained with strain FVB/N presented in the scatterplot on the right. X axis represent the log2 fold change between controls and DEHP in the targets of the microRNAs, values are averaged in case of multiple targets targeted by the same micro-RNA. Y-axis represents the log2 fold change between controls and DEHP in the microRNAs. Gray points represent microRNAs in which the MBDseq derived fold changes expressed on the x-axis was calculated with less than 10 targets, and respectively, black points with at least 10 targets up to 99, red points with more than 100 targets. Points with a star represent two-folds significantly differentially methylated microRNAs promoters at the genome-wide scale (p-values < 2.2*10^−6^, log2(FC) >1 or < -1). For all stars associated points, the short micro-RNAs symbols are written. 4 different micro-RNAs and their targets were consistent between both backgrounds and the symbol appears in bold. They are listed with the mention of the method used for the characterization of the targets and the gene symbol of their targets. ***Mmu-miR-130a-3p*** (2 targets), *Meox2* [Western blot & RT-qPCR], *Zfpm2* [Luciferase reporter assay//Northern blot//Western blot]. ***Mmu-miR-486-5p*,** (3 targets), *Foxo1* [Luciferase reporter assay//Western blot;qRT-PCR;Other], *Pten* [Luciferase reporter assay//Western blot;qRT-PCR;Other], *Pax7* [Immunocytochemistry//Luciferase reporter assay//Microarray//Western blot]. ***Mmu-miR-328-3p*** (2 targets), *Bace1* [Luciferase reporter assay//Northern blot//EMSA], *Bace2* [Luciferase reporter assay]. ***Mmu-mir-17-5p*** (353 targets, method is sequencing otherwise mentioned), *1600012H06Rik*, *1700052N19Rik*, *2510009E07Rik*, *2810055G20Rik*, *4930506M07Rik*, *4931406C07Rik*, *6030458C11Rik*, *Abca1*, *Acap2*, *Acvr1b*, *Adam10*, *Adam17*, *Adam19*, *Adcyap1r1*, *Aff4*, *Aifm1*, *Ajuba*, *Akt3*, *Aldh6a1*, *Alkbh5*, *Amot*, *Ankhd1*, *Ankrd17*, *Ankrd29*, *Ankrd9*, *Anp32a*, *Ap3d1*, *Aplp2*, *App*, *Appl2*, *Arap2*, *Arhgef9*, *Aspscr1*, *Atf2*, *Atxn3*, *B3galt2*, *B4galnt1*, *Bcl2l11* [Immunofluorescence//Immunohistochemistry//In situ hybridization//Luciferase reporter assay//Western blot], *Bmp2k*, *Bmp4* [qRT-PCR], *Brms1l*, *Brwd1*, *Btg2*, *C1qa*, *C2cd4c*, *Cacna2d1*, *Cadm2*, *Cald1*, *Camk2n1*, *Camta1*, *Capn2*, *Cav1*, *Cbx2*, *Ccdc88a*, *Ccser2*, *Cd164*, *Cdc42se2*, *Cdc7*, *Cds1*, *Celf2*, *Celsr2*, *Cend1*, *Chl1*, *Cnot6l*, *Col4a2*, *Cops2*, *Cpe*, *Cpeb3*, *Cpeb4*, *Crebrf*, *Cxcl12*, *Cyld*, *Cyth1*, *D15Ertd621e*, *D630045J12Rik*, *Dcbld2*, *Derl2*, *Dgkd*, *Dhcr24*, *Dhx36*, *Dio2*, *Dip2a*, *Dnm1l*, *Dnmt3a*, *Dpysl2*, *Dpysl5*, *Draxin*, *Dync1li2*, *Eef2k*, *Elovl6*, *Eml1*, *En2*, *Entpd7*, *Epb4*.*1l5*, *Epha7*, *Eps15*, *Erbb4*, *Etnk1*, *Extl2*, *Extl3*, *Ezh1*, *F3*, *Fam117b*, *Fam134a*, *Fam134c*, *Fam49b*, *Fat2*, *Fbxo21*, *Fbxo9*, *Fcho2*, *Fchsd2*, *Ficd*, *Flnb*, *Fn1*, *Foxp1 [Reporter assay]*, *Frmpd4*, *Gabbr2*, *Gabra1*, *Gabrb3*, *Gas7*, *Gng4*, *Gpatch8*, *Gpm6b*, *Gpr63*, *Gramd1a*, *Grb10*, *Gtf2h2*, *Gxylt1*, *Hdac8*, *Heg1*, *Hid1*, *Hsd17b10*, *Ift88*, *Igfbp7*, *Igsf3*, *Ildr2*, *Insm1*, *Islr2*, *Itgb8*, *Itm2c*, *Kbtbd8*, *Kdelr2*, *Kif21a*, *Kif5a*, *Kif5c*, *Klf10*, *Klf9*, *Klhl2*, *Klhl20*, *Klhl42*, *Kpnb1*, *Kras*, *Lcorl*, *Limch1*, *Lmbrd1*, *Lnp*, *Lpp*, *Lrch1*, *Lrp11*, *Lrrc3*, *Lrrc55*, *Lrrn3*, *Lsamp*, *Luc7l3*, *M6pr*, *Macf1*, *Map1a*, *Map2*, *Map3k5*, *Map4*, *Map4k2*, *Map7d2*, *Mapk14* [immunoblot//immunocytochemistry//Luciferase reporter assay//qRT-PCR//Reporter assay;Western blot;qRT-PCR;Other], *Mapre3*, *March6*, *March8*, *March9*, *Mcl1*, *Mcm7*, *Med17*, *Megf9*, *Mga*, *Mgll*, *Mlxip*, *Mrc1*, *Msantd4*, *Msl1*, *Mycbp*, *Mylip*, *Myo10*, *N4bp2l2*, *Napb*, *Nbea*, *Ncam1*, *Necab1*, *Nefh*, *Neurod1*, *Nf1*, *Nfe2l2*, *Nfia*, *Nipa2*, *Npat*, *Nptx1*, *Nr1d2*, *Nrip1*, *Nsg2*, *Nt5dc3*, *Nudcd3*, *Nudt18*, *Ogfod1*, *Oprl1*, *Otud4*, *Pank1*, *Papolg*, *Pappa*, *Pbrm1*, *Pcdhac1*, *Pcf11*, *Pfn2*, *Pgrmc2*, *Phlpp1*, *Pik3r4*, *Pim3*, *Plagl2*, *Plxna2*, *Polq*, *Polr3k*, *Ppap2b*, *Ppig*, *Ppp1r21*, *Ppp2r2c*, *Ppp3r1*, *Ppp6c*, *Prune2*, *Pten* [Western blot;qRT-PCR], *Ptpn11*, *Ptprg*, *Ptprj*, *Pum1*, *Pvr*, *Rab11fip4*, *Rab12*, *Rab30*, *Rab33b*, *Rabgap1l*, *Ralgds*, *Rap1gds1*, *Rapgefl1*, *Rasa1*, *Rasal1*, *Rasgef1a*, *Rassf4*, *Rb1*, *Rbl2* [Immunofluorescence//Luciferase reporter assay//Microarray//Reporter assay], *Rcan3*, *Rdx*, *Reep1*, *Rgma*, *Rnf103*, *Rnf220*, *Robo2*, *Rogdi*, *Rora*, *Rsrc2*, *Rundc3b*, *Ryr2*, *Sall3*, *Scara5*, *Scn1a*, *Scn2a1*, *Scn3b*, *Scn8a*, *Scp2*, *Sdccag3*, *Sema3c*, *Sepp1*, *Serinc1*, *Serpinb9*, *Sez6l*, *Sh3d19*, *Shh*, *Ski*, *Slc10a7*, *Slc17a7*, *Slc24a2*, *Slc35f3*, *Slc36a1*, *Slc44a5*, *Slc7a14*, *Slc7a2*, *Smim20*, *Smoc2*, *Sobp*, *Socs6*, *Sox8*, *Spag9*, *Spast*, *Srcin1*, *St6galnac5*, *Stat3* [immunoblot//immunocytochemistry//Luciferase reporter assay//qRT-PCR//Reporter assay;Western blot;qRT-PCR;Other], *Stxbp5*, *Syt1*, *Taok1*, *Taok2*, *Tbc1d12*, *Tbc1d8b*, *Tceb3*, *Tenm4*, *Tex2*, *Tfe3*, *Tgfa*, *Tgfbr2* [Luciferase reporter assay//qRT-PCR//Western blot], *Tmed8*, *Tmem230*, *Tmem64*, *Tmod2*, *Tmx4*, *Tnfrsf21*, *Tnks*, *Tnrc6b*, *Tomm34*, *Tor1aip2*, *Trim2*, *Trip12*, *Tspan9*, *Ttc14*, *Ttc9*, *Ubr3*, *Ubtf*, *Ubxn2a*, *Ugcg*, *Usp3*, *Wdr37*, *Wdr82*, *Wfs1*, *Whsc1l1*, *Xpc*, *Ythdf3*, *Zdhhc16*, *Zeb2*, *Zfand4*, *Zfhx3*, *Zfp217*, *Zfp317*, *Zfp597*, *Zfp62*, *Zfp652*, *Zfp704*, *Zfp84*, *Zhx3*, *Zic2*, *Zmat3*, *Znfx1*.
(EPS)

S3 Fig Graphical representation of CpG densities and MBDseq reads averages among the gene classes. Estimation of CpG density and averages of methylation obtained in C57BL/6J controls for the genes “other”, “miRNA”, “Olfr” and “vmn”. Medians numbers were 48 CpG sites per promoter for genes that are not belonging to the three mentioned classes, 29 for microRNAs and 9 for both Vmn and Olfr promoters. Average reads number were approximately 3 times more normalize reads within micro-RNAs promoters and 3 times less within Vmn/Olfr promoters compared to others.
(EPS)

S4 Fig Methylome, transcriptome and proteome in murine sperm. Plots illustrating developmental expression of genes in mice sperm cell subtypes in function of both methylations in promoters of sperm DNA and proteins belonging to the mice sperm proteome. The methylations levels were measured in the present study using MBD-seq data recorded in 2.2kb “promoter” probed regions in sperm cells extracted from the epididymis and the vas deferens of 8 weeks-old C57BL/6J controls mice. The genes expressions dataset of sperm cells subpopulations came from C57BL/6J males performed in a previous and independent work by Chalmel F *et al* 2007 using high-density oligonucleotide microarrays and 8–9 weeks-old mice for spermatocyte and spermatid, and 4–8 days old mice for spermatogonium. Proteins were detected in sperm cells extracted from the caudal region of the epididymis of 8 week-old swiss mice in a previous and independent work performed by Baker *et al*., 2008 using IPG strip prefractionation and LC-MS/MS. Decile expression levels were (2.68,3.6] (3.6,4.1] (4.1,4.62] (4.62,5.21] (5.21,5.81] (5.81,6.42] (6.42,7.12] (7.12,7.92] (7.92,9.03] (9.03,14].
(EPS)

S1 Table Primers sequences. Sequences of primers for bisulfite pyrosequencing and RT-qPCR.
(XLSX)
