# Supplementary material for: Prenatal Exposure to DEHP Affects Spermatogenesis and Sperm DNA Methylation in a Strain-Dependent Manner
Source: PLoS One. 2015 Aug 5;10(8):e0132136. doi: 10.1371/journal.pone.0132136 (PMC4526524; doi:10.1371/journal.pone.0132136)
Supplement: S1 Dataset — (DOCX) [file pone.0132136.s002.docx]

URL: <http://www.ncbi.nlm.nih.gov/geo/query/acc.cgi?acc=GSE67159>
